# A multi-perspective exploration of the understanding of patient complaints and their potential for patient safety improvement in general practice

**DOI:** 10.1080/13814788.2021.1900109

**Published:** 2021-04-27

**Authors:** Emily O’Dowd, Sinead Lydon, Paul O’Connor

**Affiliations:** a Discipline of General Practice, National University of Ireland Galway, Galway, Ireland; b School of Medicine, National University of Ireland Galway, Galway, Ireland

**Keywords:** General practice, quality of care, community medicine, qualitative methods, patient safety

## Abstract

**Background:**

Healthcare complaints are an under utilised source of information for safety improvement, particularly in general practice settings. Within general practice in Ireland, complaints management is dependent on individual practice policies, with little standardisation nationally, impeding their use for safety improvement. There is a need to understand factors that contribute to unlocking the potential of complaints for safety improvement in general practice in Ireland and internationally.

**Objectives:**

To explore perceptions of healthcare complaints of general practitioners, practice nurses and managers, medico-legal professionals, and policymakers.

**Methods:**

Participants were recruited using convenience sampling. Interviews were conducted from November 2019–May 2020, based on a semi-structured interview guide. Data were then transcribed and analysed using content analysis. An iterative process was applied to identify emerging themes from the interviews.

**Results:**

A total of 29 participants (19 female, 10 male) were interviewed. Three themes emerged from the analysis, ‘why patients submit complaints’, ‘management of complaints’, and ‘impact of complaints’. Subthemes included ‘barriers and facilitators to complaining’, ‘practice processes’ for complaints management, and ‘impacts on staff’ of complaints, among others.

**Conclusion:**

There is a lot to be learned about how individuals experience complaints, however, this study adds to existing knowledge. The findings from this study can be used to tackle challenges facing complaints management in general practice, including the barriers to complaining for patients and the negative impacts of complaints on the staff, and can also help to build on positive aspects of complaints such as the desire for systemic change among interested parties.

KEY MESSAGES

- Supports are needed for healthcare providers to counteract negative impacts of complaints.
- Standardised management and analysis would ensure healthcare complaints are utilised to improve patient safety in general practice.
- Patients must be encouraged to contribute to patient safety improvement through feedback such as complaints.

## Introduction

Healthcare complaints are typically defined as expressions of dissatisfaction, usually in a formal letter, regarding care provided by the health service or a healthcare provider [1]. Recent research has shown that patients have a privileged insight into the healthcare system, and can identify issues that staff or members of the organisation cannot [2]. A patient complaint is an indicator of a certain level of dissatisfaction, requiring attention from healthcare providers [3]. Patient complaints about healthcare experiences are increasingly being seen as learning opportunities to improve patient safety and quality of care [4,5].

Although relatively common, complaints are an under utilised source of information for safety improvement [6]. One recent study examined complaints made about an Irish out-of-hours service and found a total of 298 complaints out of 303,085 consultations [3]. It is evident, therefore, that healthcare complaints in general practice settings could be exploited further for patient safety improvement, in a similar manner to what is beginning to occur in secondary care [5].

A recent systematic review of studies examining complaints in general practice indicated a need for greater understanding of the variables that are crucial to unlocking the potential of patient complaints for safety and quality improvement [7]. These variables include the motivation of patients in making complaints, the potential positive and negative impacts of complaints in general practice, and how open general practitioners and other healthcare providers are to receiving and engaging with complaints [7]. Therefore the purpose of this study is to engage with stakeholders to explore the attitudes towards, and perceptions of, these aspects of complaints in general practice. It is intended that this will support the use of healthcare complaints in quality and safety improvement in the general practice context. This study used a qualitative approach to address the following research questions:

1. How do stakeholders in general practice perceive, experience and manage patient complaints?
2. What impacts do complaints have in general practice?
3. What are the perceived patient motivations for submitting complaints?

## Methods

### Design

This is a qualitative, semi-structured interview study. The study is presented in accordance with the CORE-Q guidelines for qualitative research [8].

### Context

This study was conducted in general practice in the Republic of Ireland. In Ireland, General Practitioners (GPs) work in single-handed practices, group practices, or primary care centres. Patients typically pay privately to attend a GP but some patients with special circumstances (e.g. chronic health conditions, advanced age, low income) attend their GP without paying a fee, on the public system. When making complaints about their GP, private patients must complain to the practice or co-operative, and/or to the Irish Medical Council. Public patients may also complain to the practice or co-operative and/or the national Health Service Executive (HSE).

### Participants and recruitment

GPs and people with roles in the complaints process (i.e. medico-legal professionals, complaints policymakers, practice managers and practice nurses) were recruited using a combination of convenience and snowball sampling. In the context of this study, medico-legal professionals are lawyers working at a company that provides GPs with medical indemnity cover, and practice managers are responsible for the daily operations of a GP office including staffing, scheduling, and patient complaints. Recruitment involved advertisements circulated *via* social media (e.g. Twitter), recruitment emails sent to staff in the national complaints team in the HSE, and emails sent to general practitioners in the local area. Participants were recruited throughout the duration of the research study until each of the target groups were represented. Everyone who was approached for participation from these groups took part in the study. Efforts were made to recruit patients who have made complaints about general practice, with six patient advocacy groups contacted, however, no responses were received.

### Procedure

Participants were interviewed by a female masters-level researcher (EOD), who was a PhD candidate at the time, with previous experience in conducting semi-structured interviews. The researcher was acquainted professionally with 13 of the participants, and had no prior relationship with the other 16 participants. The researcher had existing knowledge and assumptions about general practice complaints, having conducted other related research. Interviews were conducted either one-to-one in person or *via* telephone, between November 2019 and May 2020. Due to the onset of the COVID-19 pandemic, any interviews from March 2020 onwards were conducted over the telephone. Participants completed a consent form prior to taking part in the research, and knew the aims of the researcher in conducting this study.

A semi-structured interview guide (Supplemental Online Material 1) was used to structure the interviews. The guide was developed based on findings from a recent systematic review on complaints which identified patient motivations for complaining, the process of managing complaints, and the impact of complaints for learning as key priorities for research [7].

The interviews were audio recorded and subsequently transcribed by one author (EOD) who ensured that all identifying information was removed. Recordings were deleted following transcription. The transcripts were stored on a secure computer and hard drive within the researcher’s locked office on the National University of Ireland Galway (NUIG) campus. Where possible, transcripts were returned to participants for comment and/or correction.

### Analysis

Deductive qualitative content analysis was used to make meaning of the interviews [9]. This process involved familiarisation with the data, line-by-line coding, grouping of codes into hierarchical themes and subthemes, and reporting [10]. The coding scheme was based loosely on the interview guide, while also allowing for new themes to emerge from the data [9]. A sample of interviews (*n* = 7, 24%) was initially coded individually by the three authors (EOD, SL, and POC). Following this, the codes were grouped and synthesised into themes and subthemes through discussion, and by consensus, between the authors. Following this process, one author (EOD) analysed a further sample of interviews (*n* = 7, 24%). The three coders then deliberated again on the coding framework, and any new themes or subthemes which had emerged from the second round of coding were included. At this stage, NVivo 12 was used to manage the data. The remaining interviews were coded by one author (EOD), and no new themes emerged from the data at this point, indicating that a final framework had been reached. This final framework was discussed by the three authors, and following this process, the data were written up and reported by one author (EOD).

### Ethics

Ethical approval was obtained from the National University of Ireland Galway (NUIG) Research Ethics Committee (*19-Aug-15; Amend 2002*).

## Results

### Participant demographics

A total of 29 participants (19 female, 10 male) were interviewed. Participants included GPs (*n* = 13), practice nurses (*n* = 3), practice managers (*n* = 2), medico-legal experts (*n* = 4), and health service policymakers (*n* = 7). The interviews ranged from seven to 36 min in duration (mean= 19.3 min).

### Coding framework

**Figure 1.**
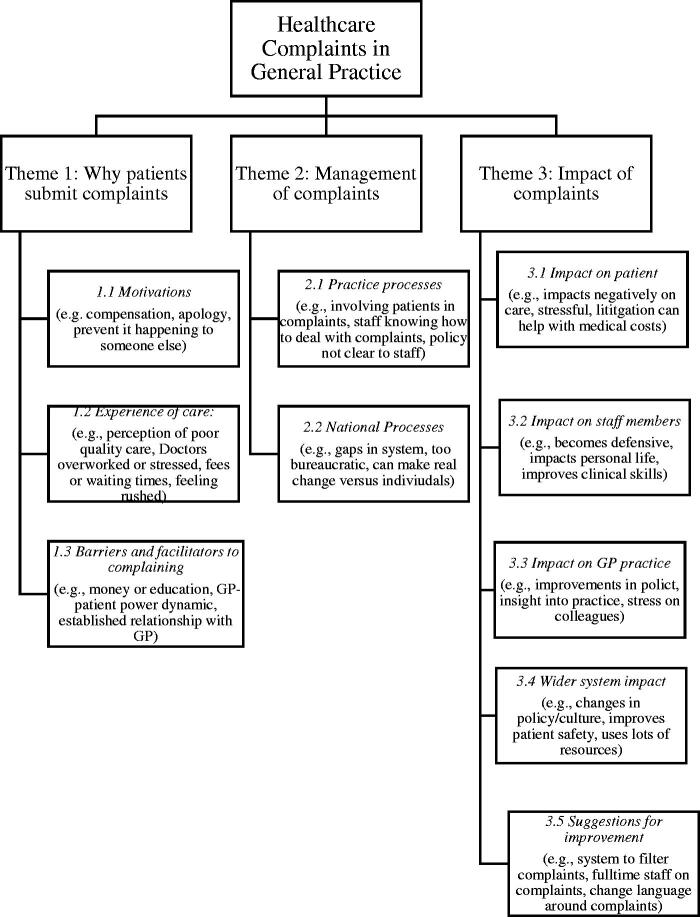
Breakdown of themes and subthemes.

The framework which emerged from the analysis consisted of three overarching themes, each with a number of related subthemes. The final themes are presented in Figure 1 below. The themes which emerged from the data analysis were: ‘why patients submit complaints’, ‘management of complaints’, and ‘impact of complaints’.

#### Theme 1: Why patients submit complaints

The first theme which emerged from the interviews was ‘why patients submit complaints’. Participants from different professional backgrounds discussed their experiences of the factors contributing to patient complaints. Three subthemes were explicated: ‘motivations’, ‘experience of care’, and ‘barriers and facilitators to complaining’. Exemplar quotes illustrating this theme and its subthemes can be found in Table 1.

**Table 1.** Exemplar quotes from Theme 1 ‘Why patients submit complaints’ and subthemes.

**Theme** *Subtheme*	No (%) of participants	Exemplar quotes
**1. Why patients submit complaints**		
*1.1 Motivations*	13 (45%)	*‘I think it is an exercise in sounding out whether they have a case for litigation or not’* (Medico-legal expert 3) *‘certainly some* [patients] *want the money back that they spent on the medication say, and you know sometimes people have very limited incomes’* (GP 1).
		*‘most people don’t really want to take you to court… they just want to make sure it doesn’t happen again to other people’* (GP 12).
*1.2 Experience of care*	23 (79%)	*‘perhaps they might feel that they weren’t treated well… you know in terms of what the treatment should have been or was’* (GP 3).
		‘*I felt the doctor was rude to me, dismissive of me, the nurse was dismissive of me, rude to me’ PP16 (GP).*
		*‘I touched on lack of resources and I think that’s a huge driver in complaints’.* (Policymaker 1).
*1.3 Barriers and facilitators*	18 (62%)	*‘more likely to complain, middle class people more than the poorer strata of society’* (GP 5).
		*‘because I suppose of the power differentials between health professionals and patients, they don’t tell you if something’s going wrong’* (Practice nurse 1).
		*‘I think as time has gone on there’s more empowerment of patients’* (GP 6).
		*‘you’re talking about small communities, people actually know who their GP is, and you know and you’re likely to meet them at mass or in a social context as well’* (Practice nurse 1).
		*‘that disappointment that might not have been complained about in-hours will be complained about in out-of-hours because it’s a strange clinician in a faceless institution’.* (GP 5).

##### Subtheme 1.1: Motivations

Patient motivations for complaining were discussed by a number of the interviewees (*n =* 13, 45%). In some cases, patients were perceived as looking for financial compensation when they complain, or as having a desire to vent or express their annoyance at the individual. In some instances, participants acknowledged that the personal circumstances may require patients to be motivated by money. However, others considered patient complaints to be made for the good of others (Table 1).

##### Subtheme 1.2: Experience of care

Patient perceptions of quality of care, along with miscommunications and misunderstandings around patient expectations, often resulted in complaints, and were discussed by 23 participants (79%).

Other issues in this subtheme related to respect and patient rights, with participants describing patients who say the doctor and other staff members were rude to them, and institutional processes, where factors such as cost, waiting times, or limited resources were seen as having contributed to a complaint (Table 1).

##### Subtheme 1.3: Barriers and facilitators to complaining

Some participants (*n =* 18, 62%) described how certain contextual and systemic factors can either facilitate or impede patient complaints. Affluent, highly educated patients were considered more likely to complain than those from lower socioeconomic backgrounds.

GP and practice nurse participants frequently brought up the imbalance of power between patients and GPs as something which can serve as a barrier to complaints. Historically, patients may have hesitated to challenge the judgement of GPs because of the power differential between patients and physicians. It was interesting that this power imbalance was raised by a practice nurse, whose position as an intermediary between GPs and patients may contribute to their understanding of the challenges faced by patients in complaining.

However, participants also described a cultural shift away from this, with increased empowerment of patients in contemporary general practice. This cultural shift away from an imbalance of power, and towards empowering patients, was considered a facilitator of patient complaints.

Another factor implicated was the personal relationship between patients and their GP, particularly in rural or small communities. Where the GP is well known to the patient they may be less likely to complain. Accordingly, where the GP is not known to the patient they were seen as being more likely to complain (Table 1).

#### Theme 2: Management of complaints

The second theme which emerged from the interviews explored participants’ experiences of how complaints are managed in the system. This theme consisted of two subthemes: ‘practice processes’ and ‘national processes’, and can be seen in Table 2.

**Table 2.** Exemplar quotes from Theme 2 ‘Management of complaints’ and subthemes.

**Theme** *Subtheme*	No (%) of participants	Exemplar quotes
**2. Management of complaints**		
*2.1 Practice processes*	28 (97%)	*‘It’s about talking to them in the first instance I suppose and trying to alleviate it without it escalating’* (Practice nurse 3).
		*‘In the place where I work currently I haven’t come across a general standard reporting procedure for complaints’* (Practice nurse 2).
		*‘I would love to see a specific policy, SOP* [standard operating procedure] *on complaints’* (Practice nurse 2).
*2.2. National Processes*	29 (100%)	*‘like I don’t know how it’s managed nationally, or broadly’* (Practice manager 1).
		*‘I think it’s too weighted against the doctor, the doctor has absolutely no recourse to complain about a patient’.* (GP 1).
		*‘I suppose I think it’s functioning better than it has been in the past. There’s definitely renewed focus on complaints and learning from complaints’* (Policymaker 3).

##### Subtheme 2.1: Practice processes

This subtheme was discussed by almost all participants (*n =* 28, 97%). Interviewees who work or have worked in general practice settings tended to focus on the importance of having defined, explicit practice processes for managing and resolving patient complaints, and recognised the need to resolve complaints quickly and locally. However, a number of participants believed that a quick resolution was hampered by a lack of a defined protocol at a practice level. It became clear from the analysis that there is often no standardised procedure for handling complaints within individual practices, or indeed across practices nationally, despite the fact that the need for one was recognised by participants (Table 2).

##### Subtheme 2.2: National processes

All participants interviewed discussed this subtheme. However, participants had contrasting opinions on the functionality and efficacy of the national process in place for managing complaints. Practice managers in particular were unaware of the national process, with their knowledge often limited to their practice processes.

Any patient complaints that were escalated beyond the practice were then handled by the doctors themselves and their legal teams, not the administrative staff within the practice. There was a clear disconnect between those working in management and policy settings, and those working in general practice. GPs and their representatives felt that the national management process was not set up to support the healthcare practitioner, and that it was weighted against the doctor.

However, those participants with a role in the national complaints process had a more positive view of the system. They mainly discussed the variation in following the national process within the country, and how it has improved from previous iterations (Table 2).

#### Theme 3: Impacts of complaints

The final theme explores the impacts of complaints, both positive and negative, on individuals and systems within general practice. This theme was divided into four subthemes based upon who or what was impacted by the complaint, all of which are presented in Table 3.

**Table 3.** Exemplar quotes from Theme 3 ‘Impact of complaints’ and subthemes.

**Theme** *Subtheme*	No (%) of participants	Exemplar quotes
**3. Impact of complaints**		
*3.1 Impact on patient*	17 (59%)	*‘if that* [the complaint] *goes all the way, the patient is going to be up on a stand as well as the doctor, getting cross-examined, and I think patients don’t essentially realise that’* (Medico-legal expert 2).
		*‘…the impact on the patient obviously is that if they feel particularly aggrieved about something, at least they have a process in place to bring it through’* (GP 12).
		*‘it perhaps makes you feel negative about that patient or their family’.* (GP 5).
*3.2 Impact on staff member*	23 (79%)	*‘no doctor remembers any good things done, but he remembers all the bad things done, all the mistakes, and the complaints, and they’re the things that stick out in the memory, because they can be very personally very difficult, very stressful, very traumatic’* (GP 9).
		‘*I know that I have heard doctors saying, well after this happened we do something differently. So there are definitely learnings that are there’* (Medico-legal expert 3).
		*‘It would make you practice more defensively, if you had someone complain about a missed test result before then you’ll end up testing everybody for that thing and that’s probably not the right way of doing it either’* (GP 10).
*3.3 Impact on GP practice*	15 (52%)	*‘because of that incident we have completely changed our practice protocols on repeat prescribing’.* (GP 9). *‘You’ve staff who are already sort of under siege in terms of the workload, media focus, you know simply trying to work in overcrowded, difficult situations, and then on top of that you have very understandable complaints…So you have a morale issue’* (Policymaker 5).
*3.4 Impact on wider system*	17 (59%)	*‘for every [complaint] …we do highlight to the powers that be and say listen there is additional resources required here… it is put on a list for when and if we do get money*’. (Policymaker 1).
		*‘it really identifies key learnings across the system but it's coming from… the voices of our patients… and how do we turn that into action how do we turn that into change’* (Policymaker 2).

##### Subtheme 3.1: Impact on patient

Some participants (*n =* 17, 59%) mentioned the impact that they perceived complaints to have on patients. These impacts were both positive and negative, with some acknowledging it a stressful experience for patients, for example when they are asked to take to the stand in front of the medical council.

However, others highlighted that it can be a positive experience for patients when a complaints process works well, as they have a process in place to deal with something that aggrieved them.

A negative impact on patient care as a result of making a complaint was discussed by some participants. Some GPs acknowledged that while they would go to all lengths to avoid treating patients differently following a complaint, that different treatment might be given to patients seen as being prone to complaining (Table 3).

##### Subtheme 3.2: Impact on staff members

A total of 23 participants (*n =* 79%) believed that complaints can have both positive and negative impacts on those on the receiving end, whether that is personally or professionally. Doctors and staff members mentioned feeling stressed, upset, angry, and burned out as a result of experiencing a complaint, and that the experience can be ‘very *difficult, very stressful, very traumatic’* (GP 9).

In terms of their professional life, while some touched on the possibility of complaints to make improvements in a doctors’ practice, with one noting ‘*I know that I have heard doctors saying, well after this happened we do something differently. So there are definitely learnings that are there’* (Medico-legal expert 3), many others discussed the increased defensiveness of GPs practice following complaints, such as over-testing patients.

##### Subtheme 3.3: Impact on GP practice

Complaints were often described by participants (*n =* 15, 52%) as having a direct, positive impact on the policies and procedures within specific practices. For instance, one GP noted that *‘because of that incident we have completely changed our practice protocols on repeat prescribing’.* (GP 9).

On the other hand, for some, complaints had a negative impact on the atmosphere in the practice, with staff morale depleted by a combination of the complaints and understaffed working conditions.

##### Subtheme 3.4: Impact on wider system

While GP, practice nurse, and practice manager participants often discussed the impact of the complaints on the GP practice itself, the legal advisors and individuals working for larger organisations tended to focus on the impacts on the system as a whole, with 17(59%) of participants addressing this. One participant reported that: *‘for every [complaint] …we do highlight to the powers that be and say listen there is additional resources required here… it is put on a list for when and if we do get money*’ (Policymaker 1).

The importance of using the patient voice to learn and improve as a national system was emphasised, particularly by people working in policy development. However, there was a recognised gap between engagement with complaints and subsequent action. For instance, one policymaker commented that: *‘it really identifies key learnings across the system but it's coming from… the voices of our patients… and how do we turn that into action how do we turn that into change’* (Policymaker 2).

## Discussion

### Main findings

Engagement with participants in this study regarding healthcare complaints has offered insights into why patients complain, how complaints are managed, and the impacts of complaints on staff, GP practices, and the healthcare system. There is a recognised lack of knowledge and understanding to facilitate the effective use of healthcare complaints for quality and safety improvement in general practice. This understanding may be used to support changing attitudes towards healthcare complaints, to enable the utilisation of complaints for quality and safety improvement, and to facilitate patient contributions to improving their care.

### Why do patients complain?

One interesting aspect of how participants experienced complaints was their perception of why patients complain. In our study, participants were most likely to attribute altruistic motivations to patients who submitted healthcare complaints. This is a positive finding, and reflective of patients’ self-reported motivations [11]. However, some participants had negative perceptions of patient motivations for submitting healthcare complaints, and further awareness-raising that patients can often have altruistic motivations is therefore required. This would help to highlight the value of these complaints, by emphasising that not all complaints are made by patients for personal gain or to spite healthcare providers. It is vital that the healthcare service capitalises on the desire from patients to contribute to safety improvement through, for example, complaints, patient experience surveys, and informal comments [12–14]. Capturing and utilising patient feedback while being cognisant of the altruistic motivations that patients can have for providing this feedback, could help improve patient care and outcomes in a participatory, inclusive manner [15].

### Local and national complaints processes

It was clear from this study that there is a need to establish continuity between practice-level and national complaints processes. There was a clear tension between local and national processes, which is potentially inhibiting the learning from complaints. Resolution of this tension would ensure that the potential of healthcare complaints to improve quality of care is realised. GPs and practice staff emphasised that they aim to resolve complaints locally before they escalate to external, formal processes. This local resolution may be beneficial to the practice involved in the complaint because issues would be dealt with swiftly, without the involvement of external bodies [16]. However, the focus on local resolution without any sharing of knowledge at a wider systems level may be detrimental to patient safety as the learning is not disseminated to others [16]. Also, the low number of complaints received by individual practices would preclude learning on broader issues. Thus, national processes need to be implemented and streamlined to ensure local complaints can effectively feed into systems-level learning from complaints [5]. Policymakers could for example provide nationally standardised guidelines for local practices to follow when building a complaints process, and also introduce the use of a framework to analyse complaints at all levels and a centralised system to facilitate knowledge exchange. The difference in perception of how the complaints process is functioning between frontline workers and policymakers is also a barrier to the effective use of healthcare complaints, and this finding echoes international research on the difference between ‘work-as done’ on the sharp end of healthcare, and ‘work-as-imagined’ [17]. This gap in understanding must be reduced, and the system transformed into one that is unified and streamlined, in order to effectively learn from experiences [18].

### The impact of complaints

The impact of complaints was discussed in a nuanced manner by participants in this study, and has built understanding of how complaints can impact upon different levels of the health service, from the individual to the system at large. Participants’ often negative perception of the impact of complaints reflects what has previously been explored in the research [19]. Currently, the complaints system is combative, with an emphasis on blame rather than on improvement [11]. Doctors have been found to face extreme stress when they receive a complaint, with GPs reporting anger, lack of confidence in practice, and even depression in the wake of a complaint [19,20]. It is clear that work is needed on reframing complaints, and restructuring the complaints process, particularly through acknowledging the potential negative impact of complaints, supporting providers who receive a complaint, and on shifting the emphasis towards a system that fosters learning rather than seeking to punish. The opportunity could also be provided to doctors themselves to respond to complaints, which in turn could reduce their negative experiences and feelings of powerlessness when they receive a complaint. This could be particularly beneficial in the instances where their clinical judgement is that the complaint is not justified.

### Learning from complaints

Despite the fact that opinions on complaints seem to be more focussed on blame than learning, participants did discuss the learning opportunities that complaints offer. There is therefore clear potential for complaints to impact positively on healthcare providers and systems [5]. Complaints can improve patient safety and experiences of care [5], and can in turn be viewed positively by healthcare providers who value this insight into the care they provide. This positive view could also contribute to maintaining therapeutic relationships following complaints by reducing emphasis on the negative aspects of complaints, and helping practitioners experience complaints as constructive instead of combative, and means of achieving these should be explored in future work. As such, there is a need to further consider how complaints can be better used as a mechanism for improving care in general practice, and how healthcare providers and managers within the health system can be supported to identify the positive impacts of healthcare complaints on practice and policy.

### Limitations

This study has a number of limitations. First, patients are not represented as participants. It was initially intended to include patients or patient advocates as participants in the study, however it proved difficult to access this group. Every effort was made to recruit patients who had made complaints, with the researchers contacting patient advocacy groups, however no response was received. Despite this, the study provides an otherwise broad sample of stakeholders in general practice, and as one review highlights, many complaints studies have tended to focus on the patient experience alone [7]. This study provides a key insight into an alternative perspective, that of individuals on the receiving end of complaints.

Second, the breadth of the research questions may be considered a limitation of the study. Each of the themes encapsulated a wealth of data, and may have benefited from a deeper exploration. The paper was exploratory by design, and intended to capture the general experiences of a wide range of people, which it achieved, however future work could take a closer look at the individual themes to see what else could emerge from their deeper study.

Third, the representation of different professional groups within the participants for this study was not equally distributed, with more GPs interviewed than other individuals. There is the potential for bias of results here, with the GP voice over-represented in comparison to practice nurses, for example. This was a necessary result of the structure of the networks for different professional groups, as practice nurses and managers for example are often more isolated within a practice than GPs in Ireland. Future work should attempt to recruit more of these under-represented groups to ensure a balanced understanding of their experiences of complaints.

Finally, the study is limited to the experiences of individuals working within the context of general practice in Ireland. Therefore, some of the findings may be specific to this context, particularly those relating to the management of complaints within the national and local systems. Nonetheless, our findings on complaints are largely aligned with those of related studies conducted in other countries [7].

### Implications

This study has illuminated some areas which warrant focus within future research and practice contexts. First, there is no standardised way of capturing the learning from complaints at both local and national levels at the moment, with some practices or areas achieving this more successfully than others. A standardised system such as a version of the Healthcare Complaints Analysis Tool (HCAT) [21] could be applied at both a local and national level to ensure consistency in complaints analysis. This in turn could capture the learning from complaints at all levels of the health service. The HCAT is a validated, reliable tool for analysing healthcare complaints about secondary care, used to identify system level trends within the data and in turn has the potential to improve patient safety through identifying hotspots and blind-spots in care [21]. A similar tool, adapted for a general practice setting, could improve the experience of receiving complaints by structuring and standardising their analysis, and could identify similar hotspots and blind-spots.

Second, research is needed to change the overall culture surrounding complaints in the healthcare system. Future research could work on interventions to change the attitudes towards complaints in general practice, or run awareness-raising campaigns to elucidate the benefits of patient complaints for all. Finally, further research could explore in greater depth how to support healthcare providers who are the subject of a complaint, to reduce the negative impact of complaints on individuals and the system at large. This in turn would benefit patient safety, by ensuring the wellbeing of healthcare providers and by emphasising the benefits over the drawbacks of healthcare complaints.

## Conclusion

Stakeholders in general practice are very aware of the potential for complaints to be used as a tool for patient safety improvement. However, work needs to be done to increase awareness of patients’ desire to contribute to safety improvement through feedback, to improve the experience of receiving a complaint for individual healthcare providers by moving from a focus on blame to a focus on learning from complaints, and to ensure the learning from complaints is standardised and shared at a national level.

## Supplementary Material

Supplemental Material - Interview Schedule
