# Supplementary material for: A multi-perspective exploration of the understanding of patient complaints and their potential for patient safety improvement in general practice
Source: Eur J Gen Pract. 2021 Apr 27;27(1):35–44. doi: 10.1080/13814788.2021.1900109 (PMC8081321; doi:10.1080/13814788.2021.1900109)
Supplement: Supplemental Material - Interview Schedule [file IGEN_A_1900109_SM5954.docx]

**Supplemental Online Material 1: Interview Schedule**

The questions I’m going to ask in this interview relate to your experiences managing or processing complaints made by patients about general practice. When discussing complaints, I’m not going to ask about specific instances or events, but rather your general experience of complaints. I will be asking questions concerning the process of complaints management, and the impact of complaints, along with the learning that can come from complaints.

I want to remind you that your responses will be confidential. Only my supervisors and I will have access to the transcript of this interview, and if any identifying details are mentioned during the interview, I will remove them from the transcript. When we write up this research study, your responses will be presented in a way that ensures you cannot be identified.

**Demographic questions:**

Position/Job title?

Years of experience?

**Interview questions:**

**Role**

1. What is your experience of complaints relating to care in general practice?

**Patient motivations**

1. What do you think are patients’ motivations to complain?
2. Why might patients not complain when they’ve experience poor quality care in general practice?

**Process**

1. Can you talk me through the process of managing a complaint? **(ASK GP ONLY)**
2. How do you think the complaints system is functioning at the moment? (prompt- for the patient, for the system, for the doctor)
3. What could be done to encourage the resolution of complaints at the lowest level/informally?
4. How could the complaints process be improved? (prompt- what are the barriers to this?)

**Impact of & learning from complaints**

1. What impacts can patient complaints about general practice have? (Prompts – on doctors, patients, practices, system, positive or negative)
2. What is your perception of the learning that comes from complaints about general practice?

- (prompts- does learning happen?
- Examples- changes in processes within practice [i.e. relating to audits etc],
- changes in care delivery,
- changes in complaints management)
- What factor(s) might determine if learning happens?

**Other issues**

1. Do you have any other thoughts or opinions about complaints in relation to general practice that have not been covered?
